# Exploring the operations of itinerant medicine sellers within urban bus terminals in Kumasi, Ghana

**DOI:** 10.1016/j.hpopen.2023.100108

**Published:** 2023-11-18

**Authors:** Joy Ato Nyarko, Kofi Osei Akuoko, Jonathan Mensah Dapaah, Margaret Gyapong

**Affiliations:** a Department of General and Liberal Studies, School of Basic and Biomedical Sciences, University of Health and Allied Sciences, Ho, Ghana; b Department of Sociology and Social Work, Faculty of Social Sciences, Kwame Nkrumah University of Science and Technology, Kumasi, Ghana; c Institute of Health Research, University of Health and Allied Sciences, Ho, Ghana

**Keywords:** Itinerant Medicine Sellers, Bus Terminals, Ghana, Medicine

## Abstract

This paper explores Itinerant Medicine Sellers’ (IMSs) operations at loading bays within bus terminals in the Kumasi metropolis. The paper examines how the sellers negotiated access into the loading bays, how they marketed their medicines, where they sourced their medicines from, and the challenges they faced. An exploratory qualitative survey design was adopted for the study. Through convenience sampling, 18 IMSs operating within the bus terminals in the Kumasi metropolis participated in this study. In-depth interviews were conducted, audio-recorded and transcribed. The transcripts were thematically analysed. The study found that these hawkers negotiated access to the loading bays through multiple informal gatekeepers and employed direct customer engagement in marketing their medicines. It was further revealed that the IMSs sourced their medicines from both formal and informal sources for different reasons, such as affordability and informality of medicines acquisition. The participants operated outside government-prescribed regulations and faced challenges of low capital and sales revenue, poor reception by prospective clients, and government clampdown. The study concludes that medicine hawking is an illegal livelihood strategy and a public health concern. As a phenomenon outside government’s policy guidelines, it is recommended that government intensifies its clampdown activities on these hawkers and engage in public health education on the negative implications of accessing medicines from these IMSs.

## Highlights

• Informal health care services providers operate on the fringes of health systems.
• Health systems’ policies are ignored by informal itinerant medicine vendors.
• Informal itinerant medicine vendors are less educated and financially motivated.
• Formal and informal medicines sources are used by informal medicine itinerants.
• Informal itinerants who sell medicines use direct customer engagement.

## 1 Introduction

Every nation is responsible for ensuring its population’s good health and well-being as enshrined in the Sustainable Development Goals [1], [2]. Essential among the targets toward achieving good health and well-being of populations is access to quality essential health care services and safe, effective, quality and affordable essential medicines and vaccines [2]. Medicines are substances that treat, prevent, or alleviate disease symptoms [3]. They are critical to every nation’s health system and are instrumental in enhancing the health of populations by reducing morbidity and mortality, and are among the fastest-used health care components [4], [5], [6]. For example, in Ghana, drugs are estimated to constitute 60–80 % of health care costs [5].

Globally, there are many structures to regulate the production, sale and dispensing of medicines [5], [7], [8]. In resource-limited settings like Africa and Asia, the sale of medicines by owner-operated drug retail outlets are common within the limits of the states’ regulatory terms and conditions [5], [9]. In Ghana, there are licensed chemical sellers who are not pharmacists but are trained and certified to sell Over-the-Counter (OTC) medicines and chemicals [7], [10], [11]. Despite these provisions, there has been the gradual creeping-in of untrained and uncertified informal medicine sellers and dispensers who illegally operate outside state regulations [12]. Due to challenges such as cost and distance, individuals resort to these informal health care service providers [13], [14]. These informal health care sources provide more than 70 % of all the primary care services in rural India and make up a significant portion of the health care sector globally, with almost half of the studies (48 %) into such activities emanating from Sub-Saharan Africa [15].

Roever and Skinner aver that itinerant traders are among the most visible informal employment [16]. These traders sell their products at busy bus terminals and streets with slow-moving vehicular and human traffic [17]. Among them are the Itinerant Medicine Sellers (IMSs), who hawk medicines [18]. These IMSs move from place to place, such as from one bus to another, at bus terminals and in rural areas, selling their medicines and other health-related products [11], [19]. IMSs are less educated and sometimes sell substandard antibiotics, give poor prescriptions and health advice leading to irrational medicine use [12], [18], [20].

IMSs and the sale of unapproved and fake medicines are on the increase, especially in sub-Saharan Africa and Asia, and can be attributed to the formal health care system’s failure to serve the population’s health care needs [12], [21]. As Sudhinaraset et al. noted, these informal medicine sellers are interstitial and contribute to the health care system, albeit without recourse to state policies [22]. Studies in sub-Saharan Africa have primarily examined how licensed/unlicensed medicines vendors perceive their role and compliance with Regulatory Guidelines and Implications for Malaria Control [12], [23]. Antimicrobial resistance studies in Africa have also looked at the negative impact of these IMSs [18], [24], [25], [26]. Although studies have raised concern over the quality of services provided by IMSs and health care delivery in rural settings, not much is known about urban IMSs and their operations [12], [27], [28], [29]. In Ghana, the Ghana Pharmacy Council (GPC) and the Ghana Traditional Medicine Practice Council (TMPC) oversee the registration and regulation of medicine sellers. Their mandate is enshrined in the Health Professions Regulatory Bodies Act (2013) Act 857. Per the Act, itinerant medicine selling is illegal. However, these IMSs seem to have flouted this directive and are operating on their own terms. How these IMSs operate in Ghana despite successive governments’ attempts to clamp down on their activities, remains a grey area [30].

A seminal study by Oppong and Williamson explored how changes in the health care system in Ghana gave rise to medicine hawkers who operated outside government policies and regulations [31]. Concurrent use of traditional herbal medicines and orthodox medicines bought from shops and IMSs, and the perspective of the Ghanaian consumer regarding the hawking of medicinal drugs have also been researched [32], [33]. Apart from these studies and few others [34], [35], [36] which indirectly examine IMSs in Ghana, there are numerous studies examining the hawking of food and other products in general without paying attention to medicines and how these IMSs operate [37], [38], [39]. Similar studies also focus on how these traders of other merchandise other than medicines conduct their businesses and how city authorities perceive them [40], [41], [42], [43], [44].

This paper forms part of a larger study which investigates the activities of IMSs, their clients and determinants of their patronage in Ghana. However, this paper explores how IMSs negotiate entry into buses to engage with clients, how the IMSs market their medicines, the sources of their medicines and the challenges they face in their operations.

## 2 Methods

*Research Design and Study Area:* An exploratory qualitative survey design was used in this study. This research design was informed by the dearth of empirical literature on IMSs in Ghana and the need to gain insights into the operations of the IMSs and medicine itinerancy within urban setups [45]. This study was carried out at bus terminals within the Kumasi metropolis, the capital city of Ashanti Region of Ghana. Kumasi is above the equator on latitude 6° 35′ north and 6° 40′ south and longitude 1° 30′ west and 1° 35′ east. The choice of the Kumasi metropolis as the study site was informed by the region having more than half of the active labour force of the informal sector in Ghana, where IMSs operate [46], [47]. Kumasi metropolis has five sub-metropolitan areas, each with a major and satellite bus terminals equipped with multiple loading bays managed by station managers who oversee the day-to-day running of the loading bays. Bus terminals in Ghana are contested spaces where informal business activities of various kinds take place, and IMSs move from one loading bay to another selling their medicines to passengers inside buses yet to take off [31], [48].

*Population and Sampling:* The larger study from which this paper is presented took place from June 2021 to November 2022. The population in this study was all IMSs who operated within the bus terminals in the Kumasi metropolis. The criteria for inclusion as an IMS were that the individual was required to be an itinerant within the Kumasi metropolis, sell medicines (herbal, Asiatic or orthodox), and operate within any of the bus terminals in the Kumasi metropolis and above 18 years of age. Due to the mobile nature of the IMSs and the illegality of their activities, the study had to rely on convenience sampling technique to recruit the IMSs. All IMSs who were encountered and consent to participate were recruited. Eighteen (18) IMSs were recruited for this study across the five major bus terminals of the sub-metropolitan areas.

*Data Collection:* The semi-structured interviews for this paper were conducted from November 10, 2021 to January 21, 2022. The data was collected from the IMSs either inside empty buses parked around the loading bays or inside the ticketing rooms of the station managers away from the loading bay noise. In-depth interviews were done with the help of a semi-structured interview guide. IMSs who were seen to have finished attending to passengers onboard buses at the loading bays were approached by the researchers. After initial introductions, the study objectives were explained to them. The consent forms were printed in duplicates and read out loud to the IMSs. Those who could read were given the forms to read by themselves. Thumbprints or signatures of IMSs who consented to the study were collected on the consent forms and a copy given to them. In the consent forms was a request to record the interviews. Those who consented were interviewed (average length of 40 min) and audio recorded with notes taken alongside, except for four of the IMSs who declined to audio recording the interviews. Some of the IMSs approached refused to partake in the study, fearing that the researchers could be government officials who wanted to arrest them. Using the interview guide, participants were asked about their day-to-day activities. In developing the guide, the larger research question (how do IMSs operate despite government’s attempt to extirpate their activities?) was interrogated by the researchers. Preliminary literature search and review was undertaken, exposing the research team to what is already known in the area. Brainstorming session regarding potential specific questions led to a draft series of questions. These questions were pilot tested on five IMSs and analysed. Changes regarding wording and ordering of questions were made and the semi-structured interview guide was constructed. Specific questions included: i) *How do you gain access to your clients?* ii) H*ow do you market your medicines?* iii) *How do you get your medicines?*

*Data Management and Analysis:* The audio recordings from the face-to-face interviews were transcribed verbatim, appropriately labelled and imported into QSR NVivo (version 12) qualitative data analysis software. The researchers read the transcripts at least twice to familiarise with the data. The inductive coding approach espoused in Saldana's coding manual for the qualitative researcher was followed in this study [49]. The first author did the data coding and the co-authors examined the codes for accuracy and consistency. Codes were grouped into themes for subsequent analysis.

*Ethical Clearance and Considerations:* Ethical clearance was sought from the Humanities and Social Sciences Research Ethics Committee, Kwame Nkrumah University of Science and Technology, Kumasi, Ghana (HuSSREC/AP/1/VOL.1), and the Institute of Health Research, University of Health and Allied Sciences, Ho, Ghana (UHAS-REC A.2[6]21-22). Ethical considerations of informed consent, anonymity and confidentiality were adhered to.

## 3 Findings

Total of 18 IMSs participated in this study. There were 15 males and 3 females. Seven had no formal education, 2 had completed primary education and 9 had completed junior high school. Regarding the type of medicines sold, 11 IMSs sold herbal medicines only, 4 sold orthodox medicines only and 3 sold herbal, Asiatic and orthodox medicines together. The oldest IMS interviewed was 65 years old and the youngest was 33 years old. The mean age was 50.6 years with a standard deviation of 7.4 years. The themes and subthemes generated are presented in Table 1.

**Table 1:** Summary of themes.

Themes	Subthemes
Negotiating access into loading bays	Single-level access negotiation
	Multi-level access negotiation
Medicine marketing strategy	Direct customer engagement
Sources of medicine for the IMSs	Formal sources
	Informal sources
Reasons for preferring different sources of medicines	Economic reasons
	Informal nature of medicine acquisition
Challenges faced by IMSs	Government
	Poor public reception
	Low sales
Challenges mitigation strategies	Patience
	Peer counselling
	Intermittent withdrawal of services

### 3.1 Negotiating access into the loading bays by the IMSs

Two main informal access negotiation strategies were used by the medicine hawkers – single-level access negotiation and multi-level access negotiation. Some medicine hawkers only negotiated access through the station managers into the loading bays, which gave them access to the buses to engage with passengers, while others had to negotiate access through the bus drivers after the initial negotiation with the station managers. Expressing their views on who allowed them to sell in buses at the loading bays, the IMSs responded:

*“The station manager. He has the power to allow me to sell there. He can ask you to leave if he does not know you, and if you do not go to him and ask permission before selling medicine in the bus. … when I first came here, I had to go to him and explain what the medicine does and who I am before he allowed me.”* IMS_18

*“Some of the drivers are difficult. You must ask permission from them even after asking permission from the station managers. If you don’t ask them, they can come and drive you out of the bus and ask you not to enter their buses again when they come to the loading bays. So, to avoid the embarrassment, you must sometimes ask for the drivers’ permission before you enter the buses.”* IMS_12

*“Just last week, I asked permission from a driver, and he said no, I should not sell in his car. Though the station manager had agreed. I turned away and left to another loading bay.”* IMS_16

### 3.2 How IMSs market their medicines

Direct customer engagement, where sellers engaged face-to-face with their customers and other prospective clients was used by the IMSs to market their medicines. With proper introduction, that is, sellers introducing themselves and their medicines to the customers and responding to any questions that customers may have on the spot, an opportunity for marketing their products was created. These direct customer engagements gave them time and opportunity to fully explain what their medicines could do. Some of the sellers also explained that with direct customer engagement, they could give details on how they use different herbs to treat different diseases and gave advice to customers on treatment regimes. IMS_8 said:

“*I don’t have a license, so I cannot do advertisements on the radio like other people who sell herbal medicines on the radio. I talk to the bus passengers and tell them about my medicine and what it can do for them. I tell them it is very bitter, so whoever cannot drink it raw should add it to porridge and drink.”* IMS_8

IMS_17 also corroborated the above statement and said:

*“I greet the passengers and tell them about me and the medicine. I even tell them where they can find me if they come to the terminal and tell them what the medicine does.”* IMS_17

### 3.3 Source of medicines for the IMSs

The IMSs got their medicines from both informal sources such as other herbalists and sellers’ own preparation, and formal sources such as pharmacies, medicine wholesalers and licensed herbal medicine shops. The informal sources related to codes which indicated unapproved and unrecognised means of preparing and acquiring medicines for sale in contravention to what is prescribed by the FDA, TMPC and GPC. These informal sources were either the sellers’ own preparations of medicines or acquiring medicines from herbalists and other IMSs unlicensed by the state. A response from IMS_14 showed that some sellers who sold herbal medicines, formulated the medicines themselves as they had informally learnt those preparations from relatives and other knowledgeable people in herbal medicines.

*“I am the eldest of my father’s children and spent most of my years growing up with my grandfather and father in our hometown. My grandfather was a herbalist in our village. I followed him and he showed me how different herbs are used to treat different diseases.”* IMS_14

*“I get my medicine from a man in Mankranso* (a town about 35 km from Kumasi)*. He is very knowledgeable in herbal medicine. He has been preparing and selling this medicine for a long time”* IMS_5

The formal sources from which the sellers got their medicines included wholesale drug vendors and pharmacies. The wholesale drug vendors in this study included local pharmaceutical wholesalers and registered large herbal medicine shops. Though none of the sellers who procured from the formal sources mentioned licensed chemical sellers, it is assumed that some did buy from these outlets as the sellers referred to both the licensed chemical sellers and pharmacies as pharmacies.

*“I currently take them from licensed herbal medicine shops around Adum and Kejetia* (within the central business district of Kumasi metropolis)*. Sometimes I call them ahead of time so that they make them* (medicines) *ready so that when I get there, I pay for them and pick them up.”* IMS_2

*“From the ABC pharmacy. I am their customer, so they know me very well.”* IMS_4

Informal nature of medicine acquisition and economic reasons were given by the IMS for preferring either the formal or informal sources of medicines. IMS_7, who bought dewormer in bulk from a pharmaceutical wholesaler and resold it with markup, said the following:

*“It is cheaper when I buy from XYZ pharmacy. They reduce the price when you are buying in bulk, and you have your cash ready.”* IMS_7

*“They don’t ask me for anything like that* (referring to license) *before selling to me. As I said, I have been working with them for a very long time, and they know me very well. I don’t have money to register. How much money [*showing her palm, indicating she has no money*] do I make to be able to save and go and register? I cannot; where is the money?”* IMS_18

### 3.4 Challenges faced by the IMSs

Three main challenges were faced by the IMS. The challenges are with the government activities to extirpate the operations of the IMS, poor public reception, and low sales. Since all the sellers interviewed could not show proof of government license permitting them to operate, it was clear that they were operating independently of government supervision. This made the sellers very careful and critical of government officials concerned with regulating the medicines space in Ghana.

*“You know when FDA or GPC make the radio announcements, they will tell the public that we are liars and are only trying to extort money from them by selling fake medicines.”* IMS_5

*“We face disrespect from some passengers* [looking down away from the interviewer]*. They think we are poor. Because you sell medicine on buses, you are not respected. This is the business I do to take care of my children in school and my wife.”* IMS_3

IMS_5 said the following regarding how they encounter some challenges with new drivers in the loading bays:

*“I was in the car selling the medicine when the driver came and shouted at me to get down. He took my bag* [showing his bag containing the medicines from his lap and putting it by the chair he was seated on in demonstration] *and put it on the ground outside the bus. I just looked at him and told him to calm down and talk to me politely. He fumed and started saying negative things about us.”* IMS_5

*“Market is not as good as it used to be, so sometimes I do not get enough chop money from the sales I make.”* IMS_9

*“I am really affected when they make those radio announcements. When it happens like that, I am not able to make enough sales”* IMS_18

In mitigating the challenges, the IMS exercised agency by being patient, engaging in peer counselling as a group and intermittent withdrawal of services. IMS_1 said the following regarding how he managed poor reception from passengers:

*“… passengers were saying things like ‘please step back, you are spitting on me’, ‘please do not stand in front of me to sell’ and ‘you people are worrying us’. But we must be patient. We are dealing with people, and that calls for patience.”* IMS_1

*“When the association* (informal IMSs’ group) *hears that government officials will be coming around, we do not come to the bus terminal. We stay away until they leave.”* IMS_5

*“We encourage and counsel one other, and share experiences on how to handle some of the challenges we face in the terminal. You know, we must look out for one another as a group.* IMS_1

## 4 Discussion

The study found that passengers onboard the buses yet to take off from the loading bays were the primary clients of the IMSs. In Nigeria, research has shown that medicine vending inside buses is ubiquitous even though regulations forbid such activities [17]. The IMSs mostly used informal access negotiation strategies through the station managers and sometimes the bus drivers. This was because there were no formally approved access negotiation procedures for people of their kind, resulting in improvisation by the IMSs. This finding agrees with the assertion by Vuban and Eta that localising access negotiation is a general ethical principle and a workable strategy for gaining access [50]. The practice of multi-level access negotiation found in this study has been alluded to by other researchers as access negotiation through different gatekeepers [50], [51], [52]. Neelakantan et al. found that gatekeepers at multiple levels may facilitate or limit access to subjects of interest, in this case, the passengers whom the IMSs wanted to engage [52]. Some of the IMSs referred to this limitation. They indicated that despite access granted by the station managers, some drivers still refused them access to the passengers on the buses. This challenge shows that access negotiation by the IMSs through the gatekeepers within the bus terminals is not always smooth, especially where there is the need to go through multiple gatekeepers.

Mramba maintains that itinerants operate as micro-informal businesses, which is crucial in determining their marketing functions and strategies [53]. These IMSs cannot operate and market their products like the formal medicine retailers, who are duly registered and recognised [23]. There is a general consensus in the literature regarding IMSs’ direct engagement with their customers since their activities are considered illegal, preventing them from making media advertisements [17], [22], [54]. The IMSs engaged with the passengers face-to-face as a group inside the buses, introduced themselves and their medicines and attended to any queries that the clients may have. Yusuff and Wassi corroborate this finding and elucidate that IMSs’ engagement with passengers inside buses allows the sellers to respond to such questions related to dosing for children, the elderly, and pregnant women [17].

A study by Syhakhang et al. on the knowledge and perceptions of drug quality among drug sellers and consumers in Lao People’s Democratic Republic found that more than half of the sellers procured their drugs from unauthorised sources and could not tell what constitutes quality medicines [55]. The use of informal sources was found in this study as well. One could opine that these sources probably sold medicines at lower prices to the sellers so that the sellers could add their markup for profits. This is because the IMSs revealed that economic motivations influence them to buy from these sources. Again, the fact that the IMSs are not recognised by the government, the informal sources of medicines served them well than the formal sources [56]. This assertion stems from their revelation of not requiring a license to purchase from the sources where they acquired their medicines. Even from the formal sources, the IMSs were not asked of any documentation before selling to them. This act could also be argued as likely due to financial motivation on the part of the formal sources since these IMSs help them sell their medicines. A critical finding regarding the informal sources of medicines was the own preparation of medicines by the sellers. This finding agrees with the assertion that herbal medicine practitioners who prepare their own medicines without approval have contributed to too many IMSs practising in the health care system in Ghana [57]. Some IMSs also bought from other sellers who prepared their medicine without approval and license from the FDA and TMPC. Walusansa et al. have found that some herbal medicine sellers purchase their medicines from fellow herbalists [58]. As indicated earlier, economic motivation seems to direct these sellers in deciding where to source their medicines without attention to medicine quality, expiry dates and regulatory guidelines.

In the context of challenges faced by IMSs, research documents issues of evictions and relocations, but focuses less on the day-to-day struggles of these workers in trying to make a living within diverse policy environments in exclusionary or contradictory ways [16]. Challenge with the government in this study was mainly regarding the extirpation activities of the FDA and the GPC toward IMSs. IMSs’ challenge with the government has been found in other studies [17], [22], [56]. A scoping review conducted found that these IMSs serve as sources of medicines for drug abusers and so are seen by the government as a nuisance [56]. In Nigeria, researchers have averred that national regulations prohibit the open market sale of medicines on the streets and inside buses [17]. This is not different from policies in Ghana [32], [59], [60]. In Ghana, many news items by the FDA and GPC prohibit selling medicines in open spaces and inside buses [61]. These news items were referred to by the IMSs, who opined that it had implications for their activities, especially sales revenue. IMSs also face disrespect from the public who are supposed to be their clients [62]. Regarding the challenge of low sales revenue by the IMSs found in this study, Jaishankar and Sujatha contend that the incomes of itinerants are often minimal, and their sales fluctuate. The activity, therefore, is a survival strategy to make ends meet [63].

Though these challenges were faced by the IMSs, they remained in business through various coping strategies such as exercising agency in withdrawing from the loading bays and sometimes from the bus terminals when they hear radio announcements banning their activities or when they are hinted of an impending visit by government officials to the bus terminals. This form of agency exercised by the sellers is functional for their survival, as it contributed positively to their operations despite attempts by governments to root out their operations from the public space. The IMSs formed informal groups through which they counselled themselves when their operations were getting tough. It has been averred that itinerants form groups to support themselves and help them deal with the challenges that come with their activities [64]. The groups, popularly referred to by the sellers as associations, created some form of solidarity and helped them to manage their challenges. The functional nature of the group formation to the existence of the IMSs cannot be overlooked, as decisions made within the group served to motivate and, at the same time, regulate group members [65].

### 4.1 Limitations

As an empirical research, this study has some limitations. This study used qualitative methods to collect and analyse data from IMSs, making generalisation beyond the study participants challenging. The generalisation of the findings from this study should be made with caution since this study was Ghanaian-focused and centred around the activities of IMSs in Kumasi. It would be challenging, therefore, to generalise beyond Kumasi, Ghana, as other countries’ cultures and social setup may differ from that of Ghana, influencing the modus operandi of IMSs in those countries. However, the study’s findings give insight into the operation of these sellers and the social setup within which they trade.

### 4.2 Conclusion

Itineracy is an informal economic activity, used as a strategy to make ends meet. However, the turning up of IMSs has been a problem that various Asian and African governments, including Ghana, have tried to address. This study concludes that IMSs are people with low to no formal education, with most of them being males, and selling herbal, Asiatic or orthodox medicines. It is an illegal livelihood strategy and of public health concern as some of them prepare their own medicines without recourse to policies. These IMSs make use of informal access negotiation strategies and employ direct face-to-face customer engagement to market their medicines and make sales. They flout various government policies and operate informally selling medicines without license or training. These IMSs are self-regulated, operating on the fringes of the formal health care system in the country and obtaining their medicines from both formal and informal sources. As a phenomenon that occurs outside government’s policy guidelines, it is recommended that the FDA, TMPC and GPC intensify their activities to quell the phenomenon. Also, government and its agencies should engage in public health education on the negative implications of accessing medicines from these IMSs.

### CRediT authorship contribution statement

**Joy Ato Nyarko:** Conceptualization, Methodology, Writing – original draft, Formal analysis, Software, Data curation. **Kofi Osei Akuoko:** Validation, Writing – review & editing, Visualization, Supervision. **Jonathan Mensah Dapaah:** Validation, Writing – review & editing, Data curation, Supervision. **Margaret Gyapong:** Writing – original draft, Validation, Supervision, Methodology.

## Declaration of Competing Interest

The authors declare that they have no known competing financial interests or personal relationships that could have appeared to influence the work reported in this paper.

## References

[1] Lublóy Á. (2014). Factors affecting the uptake of new medicines: A systematic literature review. BMC Health Serv Res.

[2] World Health Organization. WHO | SDG 3: Ensure healthy lives and promote wellbeing for all at all ages. Sustain Dev Goals 2017. https://www.who.int/sdg/targets/en/ (accessed February 4, 2020).

[3] M. Isles What’s in a Word? Falsified/Counterfeit/Fake Medicines - The Definitions Debate. Med Access @ Point Care 2017;1:maapoc.0000008 10.5301/maapoc.0000008.

[4] Embrey M., Vialle-Valentin C., Dillip A., Kihiyo B., Mbwasi R., Semali I.A. (2016). Understanding the role of accredited drug dispensing outlets in Tanzania’s health system. PLoS One.

[5] Moh Ghana National Medicines Policy 3rd Editio. 2017 Yamens Press Limited Accra.

[6] Mousnad A.M., Palaian S., Shafie A.S., Ibrahim I.M. (2018). Medicine expenditure and trends in national health insurance funds sudan. J Pharm Pract Community Med.

[7] Afari-Asiedu S., Kinsman J., Boamah-Kaali E., Abdulai M.A., Gyapong M., Sankoh O. (2018). To sell or not to sell; The differences between regulatory and community demands regarding access to antibiotics in rural Ghana. J Pharm Policy Pract.

[8] Peñaloza B., Pantoja T., Herrera C.A., Torres-Robles R., Cid C. (2015). Pharmaceutical policies: Effects of sales and dispensing policies. Cochrane Database Syst Rev.

[9] Beyeler N., Liu J., Sieverding M. (2015). A systematic review of the role of proprietary and patent medicine vendors in healthcare provision in Nigeria. PLoS One.

[10] Gyapong M., Garshong B. (2007).

[11] Hamill H., Hampshire K., Mariwah S., Amoako-Sakyi D., Kyei A., Castelli M. (2019). Managing uncertainty in medicine quality in Ghana: The cognitive and affective basis of trust in a high-risk, low-regulation context. Soc Sci Med.

[12] Liow E., Kassam R., Sekiwunga R. (2016). How unlicensed drug vendors in rural Uganda perceive their role in the management of childhood malaria. Acta Trop.

[13] Liow E., Kassam R., Sekiwunga R. (2017). Investigating unlicensed retail drug vendors’ preparedness and knowledge about malaria: An exploratory study in rural Uganda. Acta Trop.

[14] Singh M.P., Chand S.K., Saha K.B., Singh N., Dhiman R.C., Sabin L.L. (2020). Unlicensed medical practitioners in tribal dominated rural areas of central India: Bottleneck in malaria elimination. Malar J.

[15] Das J., Chowdhury A., Hussam R., Banerjee A.V. (2016). The impact of training informal health care providers in India: A randomized controlled trial. Science.

[16] Roever S., Skinner C. (2016). Street vendors and cities. Environ Urban.

[17] Yusuff K.B., Wassi A.S. (2011). Itinerant vending of medicines inside buses in Nigeria: Vending strategies, dominant themes and medicine-related information provided. Pharm Pract (Granada).

[18] Bekoe S.O., Ahiabu M., Orman E., Tersbøl B.P., Adosraku R.K., Hansen M. (2020). Exposure of consumers to substandard antibiotics from selected authorized and unauthorized medicine sales outlets in Ghana. Trop Med Int Heal.

[19] Hamilton W.L., Doyle C., Halliwell-Ewen M., Lambert G. (2016). Public health interventions to protect against falsified medicines: A systematic review of international, national and local policies. Health Policy Plan.

[20] Liow E., Kassam R., Sekiwunga R. (2018). Understanding unlicensed drug vendor practices related to childhood malaria in one rural district of uganda: An exploratory study. J Trop Med.

[21] Jamrozik E., De La Fuente-núñez V., Reis A., Ringwald P., Selgelid M.J. (2015). Ethical aspects of malaria control and research. Malar J.

[22] Sudhinaraset M., Ingram M., Lofthouse H.K., Montagu D. (2013). What is the role of informal healthcare providers in developing countries?. A Systematic Review PLoS One.

[23] Oyeyemi A.S., Ogunnowo B.E., Odukoya O.O. (2014). Patent medicine vendors in rural areas of Lagos Nigeria: Compliance with regulatory guidelines and implications for malaria control. Trop J Pharm Res.

[24] Okeke I.N., Aboderin O.A., Byarugaba D.K., Ojo K.K., Opintan J.A. (2007). Growing problem of multidrug-resistant enteric pathogens in Africa. Emerg Infect Dis.

[25] Cockburn R., Newton P.N., Agyarko E.K., Akunyili D., White N.J. (2005). The global threat of counterfeit drugs: Why industry and governments must communicate the dangers. PLoS Med.

[26] Tadesse B.T., Ashley E.A., Ongarello S., Havumaki J., Wijegoonewardena M., González I.J. (2017). Antimicrobial resistance in Africa: A systematic review. BMC Infect Dis.

[27] Gautham M., Shyamprasad K.M., Singh R., Zachariah A., Singh R., Bloom G. (2014). Informal rural healthcare providers in North and South India. Health Policy Plan.

[28] Salako L.A., Brieger W.R., Afolabi B.M., Umeh R.E., Agomo P.U., Asa S. (2001). Treatment of childhood fevers and other illnesses in three rural Nigerian communities. J Trop Pediatr.

[29] Okonkwo A.D., Okonkwo U.P. (2010). Patent medicine vendors, community pharmacists and STI management in Abuja. Nigeria Afr Health Sci.

[30] MOH MOFA MESTI, MFAD. National Action Plan (NAP) for Antimicrobial Use and Resistance in Ghana Ghana National Action Plan for Antimicrobial Use and Resistance Ministry of Health Ministry of Food and Agriculture Ministry of Environment Science Technology and Innovation Minist. 1st ed. Accra: Ministry of Health, Ghana. 2017.

[31] Oppong J., Williamson D. (2001). Locating itinerant drug vendors in ghana’s health care system. African Geogr Rev.

[32] Azila-gbettor E.M., Atatsi E.A., Adigbo E.D. (2014). Hawking of medicinal drugs : The perspective of the ghanaian consumer. Res Humanit Soc Sci.

[33] Ameade E.P.K., Ibrahim M., Ibrahim H.-S., Habib R.H., Gbedema S.Y. (2018). Concurrent use of herbal and orthodox medicines among residents of tamale, Northern Ghana, who patronize hospitals and herbal clinics. Evidence-Based Complement Altern Med.

[34] Van Andel T., Myren B., Van Onselen S. (2012). Ghana’s herbal market. J Ethnopharmacol.

[35] Owusu A.B., Abrokwah S., Frimpong S. (2013). Analysis of the spatial and temporal dynamics of street hawking: A case study of the accra metropolitan area. J Geogr Geol.

[36] Affinnih Y.H. (1999). Drug use in greater accra, Ghana: Pilot study. Subst Use Misuse.

[37] Davis J. (2008). Selling wares on the streets of accra: A case study of street hawkers in ghana’s capital. Focus Geogr.

[38] Akuoko K.O., Ofori-Dua K., Forkuo J.B. (2013). Women making ends meet: Street hawking in kumasi. Challenges and Constraints Michigan Sociol Rev.

[39] Yeboah T., Owusu L., Arhin A.A., Kumi E. (2015). Fighting poverty from the street: perspectives of some female informal sector workers on gendered poverty and livelihood portfolios in southern Ghana. J Econ Soc Stud.

[40] Amoatemaa A. (2017). Implications of hawking activities on the kumasi campus of university of education, winneba. Br J Educ Soc Behav Sci.

[41] Forkuor J.B., Akuoko K.O., Yeboah E.H. (2017). Negotiation and management strategies of street vendors in developing countries: A narrative review. SAGE Open.

[42] Spire A., Choplin A. (2017). Street vendors facing urban beautification in accra (Ghana): Eviction, relocation and formalization. Artic – Rev Sci Hum.

[43] Amoako J. (2019).

[44] Okoye V., Sands J., Debrah C.A. (2010). The accra pilot bus-rapid transit project: Transport-land use research study. Accra.

[45] Rendle K.A., Abramson C.M., Garrett S.B., Halley M.C., Dohan D. (2019). Beyond exploratory: A tailored framework for designing and assessing qualitative health research. BMJ Open.

[46] Ghana Statistical Service. Ghana Living Standards Survey round 7 (GLSS7). Accra: 2019.

[47] Sieverding M., Beyeler N. (2016). Integrating informal providers into a people-centered health systems approach: Qualitative evidence from local health systems in rural Nigeria. BMC Health Serv Res.

[48] Steel W., Ujoranyi T., Owusu G. (2014). Why evictions do not deter street traders: Case study in accra. Ghana Ghana Soc Sci J.

[49] Saldana J. (2016).

[50] Vuban J.A., Eta E.A. (2019). Negotiating access to research sites and participants within an African context: The case of Cameroon. Res Ethics.

[51] Lata L.N. (2021). Negotiating gatekeepers and positionality in building trust for accessing the urban poor in the Global South. Qual Res J.

[52] Neelakantan L., Fry D., Florian L., Meinck F. (2022). Adolescents’ experiences of participating in sensitive research: A scoping review of qualitative studies. Trauma, Violence, Abus.

[53] Mramba N.R. (2015). The marketing communication strategies of street vendors in Dar es Salaam Tanzania. Acad Sch Res J.

[54] Abubakar K., Biambo A.A., Samaila A., Usman N., Abubakar S.B., Muhammad A. (2015). Factors responsible for proliferation of drug hawking in Sokoto Metropolis, North Western Nigeria. Basic Res J Med Clin Sci.

[55] Syhakhang L., Freudenthal S., Tomson G., Wahlström R. (2004). Knowledge and perceptions of drug quality among drug sellers and consumers in Lao PDR. Health Policy Plan.

[56] Jatau AI., Sha’aban A., Gulma KA., Shitu Z., Khalid GM., Isa A. (2021). The burden of drug abuse in nigeria: A scoping review of epidemiological studies and drug laws. Public Health Rev.

[57] Moh Policy guidelines on traditional medicine development Accra 2005.

[58] Walusansa A., Asiimwe S., Ssenku J.E., Anywar G., Namara M., Nakavuma J.L. (2022). Herbal medicine used for the treatment of diarrhea and cough in Kampala city. Uganda Trop Med Health.

[59] GoG. Health Professions Regulatory Bodies Act (Act 857). Accra: Ghana Publishing Company (Assembly Press); 2013.

[60] GoG. Traditional Medicine Practice Council Act (Act 575). Accra: Ghana Publishing Company (Assembly Press); 2000.

[61] KJ. Anane Arrest any person who sell medicines in buses and houses- Minister 2018. https://otecfmghana.com/2018/09/arrest-any-person-who-sells-medicines-in-buses-and-houses-minister/ (accessed July 17, 2020).

[62] Fonceca C.M., Keerthivasan S., Christi Anandan C.R., Lisa E K. (2022). Exploring the reasons for street hawking, challenges & quality of life of street vendors | Journal of positive school psychology. J Posit Sch Psychol.

[63] Jaishankar V., Sujatha L. (2016). A study on problems faced by the street vendors in tiruchirappalli city. SSRG Int J Econ Manag Stud.

[64] Baniboye V.O.H. (2015).

[65] Bachmann T. (2022). Functional group positions and contact behavior in problem-solving groups. Grup Interaktion Organ Zeitschrift Fur Angew Organ.

